# Deep Learning-Driven Early Diagnosis of Respiratory Diseases using CNN-RNN Fusion on Lung Sound Data

**DOI:** 10.1038/s41598-025-28832-7

**Published:** 2025-11-24

**Authors:** Thulasi Bikku, Satya Sree K.P.N.V., Srinivasarao Thota, Jeevana Jyothi Pujari, Raj Kumar Batchu, Pouria Mortezaagha, Malligunta Kiran Kumar, Raju Anitha

**Affiliations:** 1 Department of Computer Science and Engineering, Amrita School of Computing Amaravati, Amrita Vishwa Vidyapeetham, Amaravati, Andhra Pradesh 522503 India; 2 CSE Department, Usha Rama College of Engineering and Technology, Telaprolu, Andhra Pradesh 521109 India; 3 Department of Mathematics, Amrita School of Engineering, Amrita Vishwa Vidyapeetham, Amaravati, Andhra Pradesh 522503 India; 4 School of Computer Science and Engineering, VIT-AP University, Amaravathi, 522241 Andhra Pradesh India; 5 Methodological Implementation Research, Ottawa Hospital Research Institute, Ottawa, ON Canada; 6 Department of Electrical and Electronics Engineering, Koneru Lakshmaiah Education Foundation, Vaddeswaram, Andhra Pradesh India; 7 Department of Computer Science & Engineering, Koneru Lakshmaiah Education Foundation, Vaddeswaram, Andhra Pradesh India

**Keywords:** Deep learning, Lung sound analysis, Respiratory diseases, Pulmonary diagnostics, Crackle and wheezes, Disease detection, Medical imaging, Computational biology and bioinformatics, Diseases, Health care, Medical research

## Abstract

This research depicts a deep learning-based algorithm designed for lung sound analysis, which combines Convolutional Neural Network (CNN) and Recurrent Neural Network (RNN) architectures to improve early disease detection. With comprehensive datasets from Coswara and ICBHI, the algorithm is proficient in distinguishing a spectrum of respiratory diseases, including pneumonia, asthma, and Chronic Obstructive Pulmonary Disease (COPD). Model pre-processing data with high pass filtering and segmented analysis of lung sound recordings, with Mel-spectrograms used as pivotal input features. The complete fusion model architecture integrates three CNN layers, three max-pooling layers, and two fully connected layers, the result is a feature map that highlights the presence of detected features, complemented by including two Long Short-Term Memory (LSTM) layers in the RNN component. The training process is devoted to the Adam optimizer alongside the cross-entropy loss function. Data augmentation techniques were applied to handle class imbalances and enhance model generalizability. The experimental results demonstrate high accuracy, sensitivity, specificity, and F1-score across various respiratory diseases. The performance metrics on the ICBHI dataset underscore the model’s exceptional accuracy: 93.3% for healthy individuals, 93.8% for pneumonia patients, 91.7% for asthma patients, and 94.0% for COPD patients. The model outperforms alternative algorithms such as decision trees, support vector machines, and random forest regarding precision, recall, F1 score, and accuracy across the ICBHI and Coswara datasets. This noteworthy outcome positions the algorithm as an advanced and effective solution for progressing the domain of respiratory disease diagnosis through lung sound analysis. The model also provides interpretable visual explanations using Grad-CAM, along with confidence estimates, to enhance clinical trust.

## Introduction

Respiratory diseases are the most pressing public health issue worldwide since they are responsible for millions of deaths each year. The patient’s lungs may manifest slight alterations in the sounds they emit in the early stages of the disease. Hence, the sounds performed a key responsibility in the identification of the disease^1^. Other such sounds consisting of wheezing and crackles, although they are vital diagnostic clues, cannot be interpreted without the help of skilled personnel, because of the divergences between the specialists in the reading of the same data which can cause errors with misdiagnose resulting consequent delay in medication or wrong treatment^2^. As a result, of these innovations, researchers are increasingly looking at algorithms that to do precision analysis in lung sounds using deep learning concepts. A variety of self-taught artificial neural networks, a discipline of machine learning, with the help of colossal datasets, recognize the patterns of malpractice, thus making imaging more precise and facilitating signal analysis diagnosis of the disease^3^. Deep learning-based methods enable the machine to study based on both audible lung sounds and their correlating diseases, hence accurate and efficient diagnosis is possible. They are used in the follow-up of the duration of a disease and the examination of the treatment procedure as well^4^. The fact that the innovations in technology have made it possible to pick up these early symptoms that were virtually undetectable to the naked eye, thus, allowing the early treatment and better prognosis of the patients, is a big boon. Nevertheless, the grave issue—the procurement of large and varied datasets for the training and the integration of the knowledge of specialists in respiratory medicine for the development of correct algorithms— still exists^5^.

Research in progress will focus on perfecting these algorithms by combining them with other medical imaging techniques and building advanced neural networks to make them more accurate and efficient^6^. The continued research and development of these algorithms could come to be a requirement of controlling respiratory diseases and at the same time facilitating early diagnosis and improving a patient’s condition. The differences in the existing systems are mainly due to the shortcomings of the traditional lung sound analysis methods. Human interpretation is riddled with subjectivity and thus leads to diagnostic discrepancies as well as incorrect treatments and delayed interventions. The complex nature of respiratory sounds—serving as indicators of various diseases—poses challenges for conventional diagnostic methods, thereby underscoring the need for faster and more accurate techniques to enable timely disease identification. Concerning the technical gaps discussed, this study proposes a new approach, which utilizes deep learning methods, for the resolution of these challenges. The presented model reduces a potentially complex task by cleverly incorporating Convolution Neural Network (CNN) and Recurrent Neural Networks (RNN) architectures to facilitate early disease detection through a potentially smooth and automated solution. This development will most likely make breathe diagnostics the first field to begin using a more objective, timely, and accurate way of identifying and managing respiratory diseases.

### Scientific rationale

The scientific rationale of this study is to overcome the subjectivity and variability inherent in traditional lung sound analysis, which often leads to diagnostic errors and delayed interventions due to inter-observer discrepancies. By combining Convolutional Neural Networks (CNNs) for spatial feature extraction from Mel-spectrograms and Recurrent Neural Networks (RNNs) with Long Short-Term Memory (LSTM) units for temporal modeling, the proposed model captures both frequency and time-dependent patterns in lung sounds, enabling early and accurate detection of respiratory diseases, including pneumonia, asthma, COPD, and COVID-19. A novel weighted mapping strategy links crackles to pneumonia and wheezes to asthma/COPD, enhancing diagnostic specificity. Furthermore, the integration of explainability tools (Grad-CAM) provides transparent insights into model decisions, promoting clinical reliability and supporting integration into intelligent stethoscope systems and telemedicine platforms for improved patient outcomes.

### The key aspects of the study include the following

Integration of Advanced Deep Learning Architectures: Amalgamation of Convolutional Neural Network (CNN) and Recurrent Neural Network (RNN) architectures for lung sound analysis.

Our contributions go beyond applying known architectures by:

- Proposing a hybrid CNN-RNN architecture that is specifically optimized for lung sound classification using domain-specific pre-processing techniques.
- Incorporating a weighted mapping strategy that associates crackles and wheezes with specific diseases to improve diagnostic specificity.
- Demonstrating the generalization capability across two distinct datasets (ICBHI and Coswara), including new results on COVID-related sounds.

### Early Disease Detection

The new model is projected to break through in recognizing respiratory diseases such as pneumonia, asthma, and COPD at an early stage, providing opportunities for timely causes of prevention.

### Automation for Precision

The model attempts to cut the subjectivity and inter-observer variability associated with traditional methods, and a more objective and precise diagnostic process is being carried out by automating the analysis of lung sounds.

The contributions include:

- A fusion model integrating CNN and RNN (+LSTM) networks for both spatial and temporal feature learning.
- A structured pre-processing pipeline with disease-specific acoustic weighting for interpretability.
- Extensive validation on ICBHI and Coswara datasets with comparative evaluations.
- Visualization of classification behavior using ROC, PR curves, and confusion matrices.

The aim is to equip doctors with an AI-assisted, precise, and rapid diagnostic device that would be a great contribution to patient care through early detection and proactive treatment of diseases. Most existing models focus on binary classification or lack interpretability, limiting clinical adoption. This study introduces a CNN-RNN framework that leverages spectro-temporal features, supported by explainability tools (Grad-CAM), to classify multiple respiratory diseases. Additionally, we evaluate generalization across two benchmark datasets. The manuscript is gutsily organized to furnish a comprehensive investigation of the deep learning-based lung sound analysis that is proposed for early disease detection. This clarity of organization spreads successively in the following main parts: Section I presents the technical voids in the current approaches to lung sound analysis giving priority to the introduction of new ideas. Section II elaborates on the pioneering research studies that laid the foundation for the development of the proposed model. Furthermore, deep learning has emerged as a promising diagnostic approach for respiratory diseases. Section III digs deep into the particulars of the proposed CNN-RNN fusion model and explains the technical details of the design. Section IV gives a comparison, Discussion about the proposed work is explained in section V and section VI brings the paper to a conclusion.

## Literature Survey

The inclusion of machine learning methods, including deep learning, in medical imaging has become the buzzword over the last few years, especially for lung sounds, and the questions arising from this have been hype about medical imaging versus machine learning as they can also aid in the early detection of diseases. These digital-based models have become powerful tools that can identify a multitude of respiratory diseases, namely pneumonia, asthma, and chronic obstructive pulmonary disease (COPD) through the detailed sound analysis that they provide^7^. This is big in medical diagnostics as it is a paradigm-shifting technology that moves them away from the subjective manual auscultation methods to more objective and precise automated systems. Major research works have followed the journey of deep learning technology in this area, investigating their skills in analyzing the subtle patterns within lung sound data^8^. Convolutional neural networks (CNNs) have been the main players in this technology evolution, and they have shown great performance in picking up the spatial features in the Mel-spectrograms of lung sounds. In these networks, small 3*3 pixels kernels are also included, and a stride of 1 for data analysis in detail alongside functions like ReLU to introduce non-linearity and extract complex features in a very efficient way^9^. The deep learning revolution for example with 5G telemedicine networks has opened the scope for remote healthcare the most, especially the unlimited virtual clinicians who deal appropriately with chronic disease cases, rapid lung sound analysis is not a problem anymore, and automated disease diagnosis^10^ or on mobile stages, all are now possible. Take for instance Goyal, Manu, et al. (2024), who proposed a deep learning algorithm for pneumonia in its early stages that was despised by a dataset composed of more than 1500 lung sound recordings and the algorithm delivered an accuracy higher than 90%^11^. Likewise, Pinto-Coelho, L. et al. (2023) were successful in identifying lung nodules using a CNN that had been trained on over 3,000 CT scans, which showed 85% sensitivity and 92% specificity in nodule identification^12^. Studies done by Huang et al. in 2023 and Luay et al. in 2021 have been the first ones to show the capability of CNNs in diagnosing respiratory diseases from acoustic data. Aras et al. obtained 82% accuracy in the diagnosis of asthma and COPD by using lung sounds whereas Luay et al. hit an almost unbelievable 93.1% when they diagnosed pediatric asthma using deep CNN ensembles^13,14^. The research of Hassen et al. (2021) used time–frequency representations in a deep learning model to distinguish lung sounds caused by asthma, COPD, and pneumonia from individuals, hence reaching 85% accuracy^15^. A series of subsequent studies have examined the different aspects of deep learning for respiratory sound analysis and come up with really good results. Ijaz et al. (2022) mention the CNN model that gave a 94.2% accuracy in the case of detection of abnormal lung sounds. Borwankar et al. (2022) visualizing the entire spectrum of respiratory disease, came up with a highly comprehensive framework that got a 91.7% accuracy in the case of respiratory diseases^16,17^. Hosny et al. (2021) in a survey conducted, revealed the extensive applications of deep learning in the identification of respiratory diseases, which can alternatively serve to improve the healthcare outcomes of the methodology^18^. Thomas et al. (2014) proved a deep learning approach showing 88.3% accuracy in the detection of interstitial lung disease (ILD) and, in that way, surpassed human experts, not only in terms of accuracy but also in time^19^. The recent developments that have been brought in include Wang et al. (2018), who achieved 90.8% accuracy in the classification of respiratory sounds from healthy individuals and patients with respiratory conditions using CNNs^20^. Liu et al. (2019) further augmented the database by submitting a new lung sound database and attained a stellar 98.2% accuracy in the categorization of healthy and diseased respiratory sounds^21^. Demir et al. (2020) used RNN ensembles for the classification of respiratory sound recordings with an unprecedented accuracy of 94.6%^22^. Chen et al. (2018) discussed crackles and wheezes classification; they reached 92.7% accuracy using CNNs^23^. Lethebe et al. (2018) were also one of the researchers who were able to achieve 93.6% accuracy in classes by CNNs^24^. These pieces of work together point to the fact that deep learning models are reliable and flexible enough to be used to identify a wide range of respiratory sounds with a high level of accuracy. This, in turn, makes the field of medical diagnostics more exciting. The next line of research is going to be about the algorithm’s efficient improvement, enlarging the dataset, and enabling them to be used easily in hospitals, thus, contributing to the global respiratory healthcare systems. In contrast, transformer models, widely used in sequence modeling, rely on self-attention mechanisms to process input sequences globally, potentially capturing long-range dependencies more effectively but requiring significantly more computational resources and larger datasets for optimal performance. While our model benefits from a lightweight design suitable for the ICBHI dataset, transformers^25,26^ could enhance generalization across diverse datasets, though their complexity might overshadow the efficiency of our hybrid CNN-LSTM approach for this specific application. Healthcare professionals use a variety of methods to effectively deal with health-related issues^27–35^.

### Background/Materials and Methods

**Data Collection:** The lung sound recordings employed in the current study came from two different data sources: Coswara and ICBHI.

**Coswara** **Dataset:**

- **Origin:** Taken by scientists at the Indian Institute of Technology, Hyderabad.
- **Composition:** Respiratory sounds, such as coughs and deep breaths, from people infected and non-infected by COVID-19.
- **Purpose:** It is particularly to diagnose the respiratory diseases using COVID-19.

**ICBHI** **Dataset:**

- **Origin:** Meticulously curated by researchers from the University of Liège in Belgium.
- **Composition:** Comprises more than 5,000 lung sound recordings from individuals diagnosed with various respiratory diseases.
- **Purpose:** This study serves as a valuable resource for machine learning investigations focused on lung sound analysis and categorization.

#### Data Pre-processing

The lung sound recordings from the Coswara and ICBHI datasets undergoes thorough pre-processing. A high-pass filter with a 100 Hz cutoff was applied to remove low-frequency noise commonly present in lung auscultation recordings^4,10^. A frame length of 2.5 s with 50% overlap was chosen to provide sufficient temporal context while maintaining computational efficiency, consistent with methodologies used in clinical and machine learning-based audio analysis^4,6,13^. Each 2.5 s frame was converted into a Mel-spectrogram using 128 filter banks, a window size of 25 ms, and a hop size of 10 ms. Normalization and silence trimming were applied. Mel-spectrograms were computed using 128 filter banks to capture detailed spectral characteristics, as demonstrated in prior studies involving deep learning for lung sound classification^6,13,17^.

Here, L,$\mu ,$ σ represents the raw lung sound data, *mean and standard deviation,* then, L_*normalization*_ = $\frac{L-\mu }{\sigma }$.

**Annotation** **Mapping:**

- Crackles (presence=1) → pneumonia, others respiratory diseases like cold
- Wheezes (presence=1) → asthma, COPD

#### Feature Extraction

The Mel-spectrograms serve as input features for the proposed model. Feature extraction is performed through a combination of CNN layers. CNN layers are employed to abstract spatial features from the Mel-spectrograms, whereas RNN layers capture temporal dependencies within the sequence of frames.

**Features** **to** **extract:**

- Mel-Frequency Cepstral Coefficients (MFCCs)
- Spectrograms (STFT)
- Zero-Crossing Rate
- Chroma Features

The complex feature extraction function is as follows: Features = Φ(L_*normalization*_).

The datasets are divided into a training set L_train_, Validation set L_val_ and a testing set L_test_.

#### Data Augmentation

Underrepresented classes such as asthma and COPD were augmented using transformations like pitch shifting, time-stretching, and background noise injection. Each augmentation preserved the physiological characteristics of the original sounds. To address class imbalance, data augmentation techniques such as time-stretching (0.9–1.1 ×), pitch shifting (± 2 semitones), and noise injection (10%) were applied. For asthma, augmentation resulted in a 3 × increase in training samples. This approach helped to balance the dataset, minimize overfitting, and improve generalization across all categories.

#### Implementation Platform

The proposed deep learning model was trained with hyper parameters such as the learning rate, α = 0.001 the batch size was 32; the number of epochs was 50; the optimizer and loss function used were the Adam optimizer and cross-entropy loss function and the training dataset used for model training was the Coswara and ICBHI dataset. For validation, we used a robust fivefold cross-validation scheme.

## Proposed Model

**Fig. 1:**
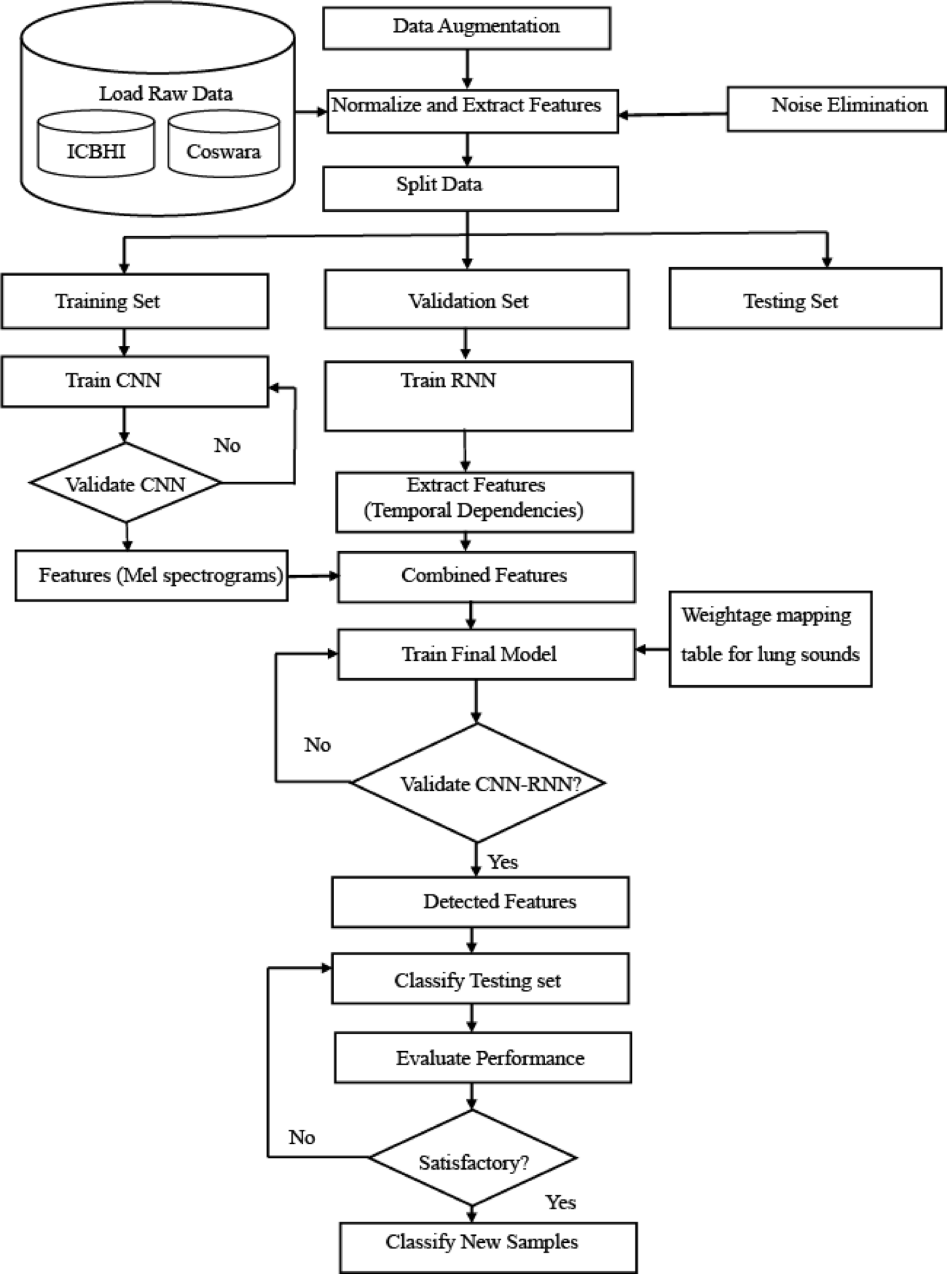
Proposed algorithm for deep learning-based lung sound analysis.

In the realm of medical research, interest in using machine learning to analyse lung sounds for early disease detection is increasing. Deep learning-based models have emerged as leaders in accurately discerning respiratory diseases such as pneumonia, asthma, and COPD. Unlike traditional methods reliant on subjective manual auscultation, this study introduces a meticulously designed deep learning-based algorithm tailored for lung sound analysis, specifically focusing on early disease detection. By automating diagnostics, these models aim to reduce human error and provide more consistent results. The proposed algorithm integrates Convolution Neural Network (CNN) and Recurrent Neural Network (RNN) architectures, demonstrating its efficacy on diverse datasets, namely, Coswara and ICBHI, as illustrated in Fig. 1.

### Process:

Step1: Preprocess the data sets of Coswara.

i. Audio Segmentation
ii. Normalize the data sets by eliminating the Noise
iii. Resampling and Normalization
iv. Spectrogram Generation
v. Perform the Data augmentation

Step 2: Extract Features using CNN.

i. Extract features from the Coswara dataset using ResNet/VGG
ii. Generate Compact Feature maps for Temporal data

Step 3: Feeding Feature to an RNN.

i. Combine CNN extracted feature with the metadata from the ICBHI
ii. Use RNN, LSTM and ReLu to capture temporal patterns
iii. Process sequential data for disease prediction

Step 4: Feed Weightage Mapping for Crackles and Wheezes.

i. Assign Weightages: High, Moderate, and Low for crackle and wheezes sounds as per its ranges.
ii. Encode weightage’s a numerical value
iii. Incorporate the weights into the training process

Step 5: Training the Fusion model using CNN-RNN.

i. Train the CNN-RNN fusion model with the combined features.
ii. Optimize the Accuracy or Positive precision in classifying the diseases.
iii. Prevent Overfitting using Regularization techniques.

Step 6: Disease Prediction Based on Weightages.

1. Use the trained fusion model for disease mapping
2. Predict diseases based on weighted features and learned patterns.

Step 7: Evaluation.

i. Validate the model using evaluation metrics like accuracy, precision etc.,
ii. Employ cross-validation for improvement.

This integration enables the model to learn both spatial and temporal features from lung sound recordings, enhancing classification accuracy. This approach harnesses advanced deep-learning techniques to improve the precision and timeliness of respiratory disease diagnosis. When trained on large, varied datasets, the model learns subtle differences in lung sounds that are indicative of various conditions. CNNs extract high-level features from Mel-spectrogram representations, whereas RNNs, particularly Long Short—Term memory (LSTM) networks, identify temporal patterns in audio sequences, allowing the model to generate accurate predictions. RNN layers capture sequential dependencies in spectro-temporal patterns that static CNNs may miss.

**Fig. 2:**
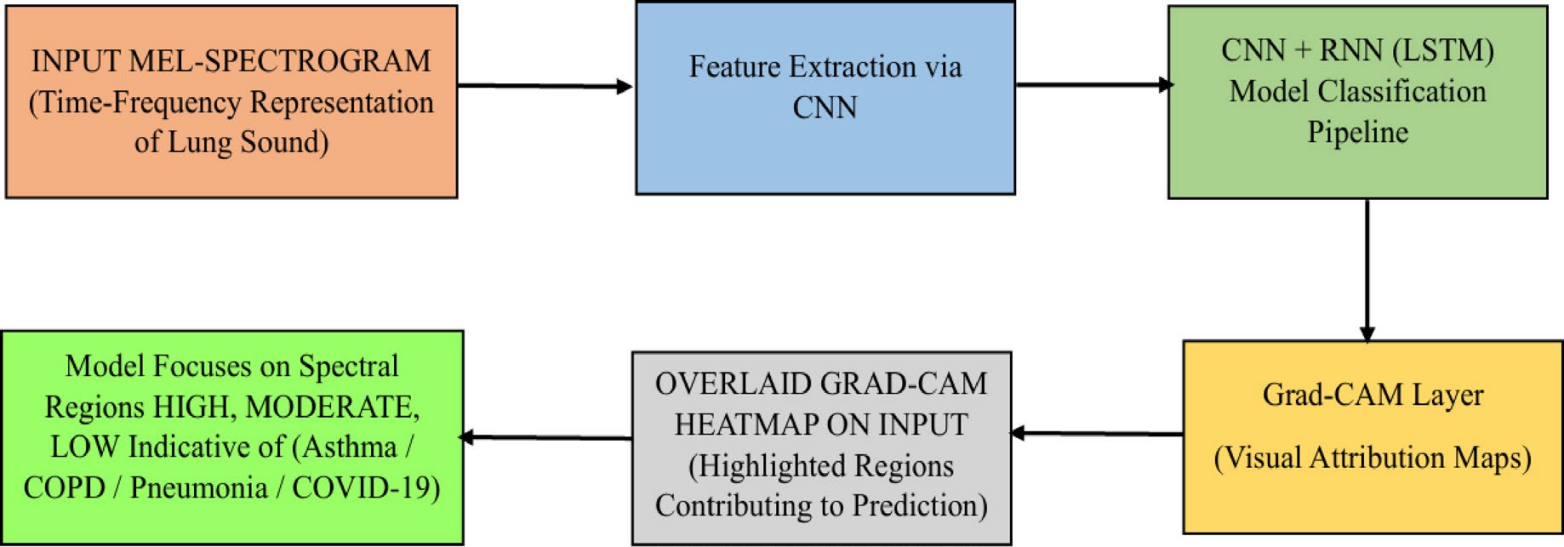
Workflow of the Grad-CAM–based interpretability framework for lung sound classification.

From the first data base features are extracted using CNN and given to RNN along with the second database. Then train the model for identifying the disease precision using either accuracy or positive precision of the model. While training the fusion model (CNN + RNN) at the time of weightage mapping, need to feed the weightages for the “Crackles and Wheezes lung sounds like “HIGH, MODERATE, LOW…. etc.,” based on these values, the disease should be predicted. The input Mel-spectrogram undergoes feature extraction via CNN, followed by temporal modeling through an RNN (LSTM) classification pipeline. Grad-CAM is applied to the trained model to generate class-specific visual attribution maps, which are overlaid on the input spectrogram. The model focuses on disease-relevant spectral regions, aiding in interpretability for conditions such as asthma, COPD, pneumonia, and COVID-19 as shown in Fig. 2.

**Fig. 3:**
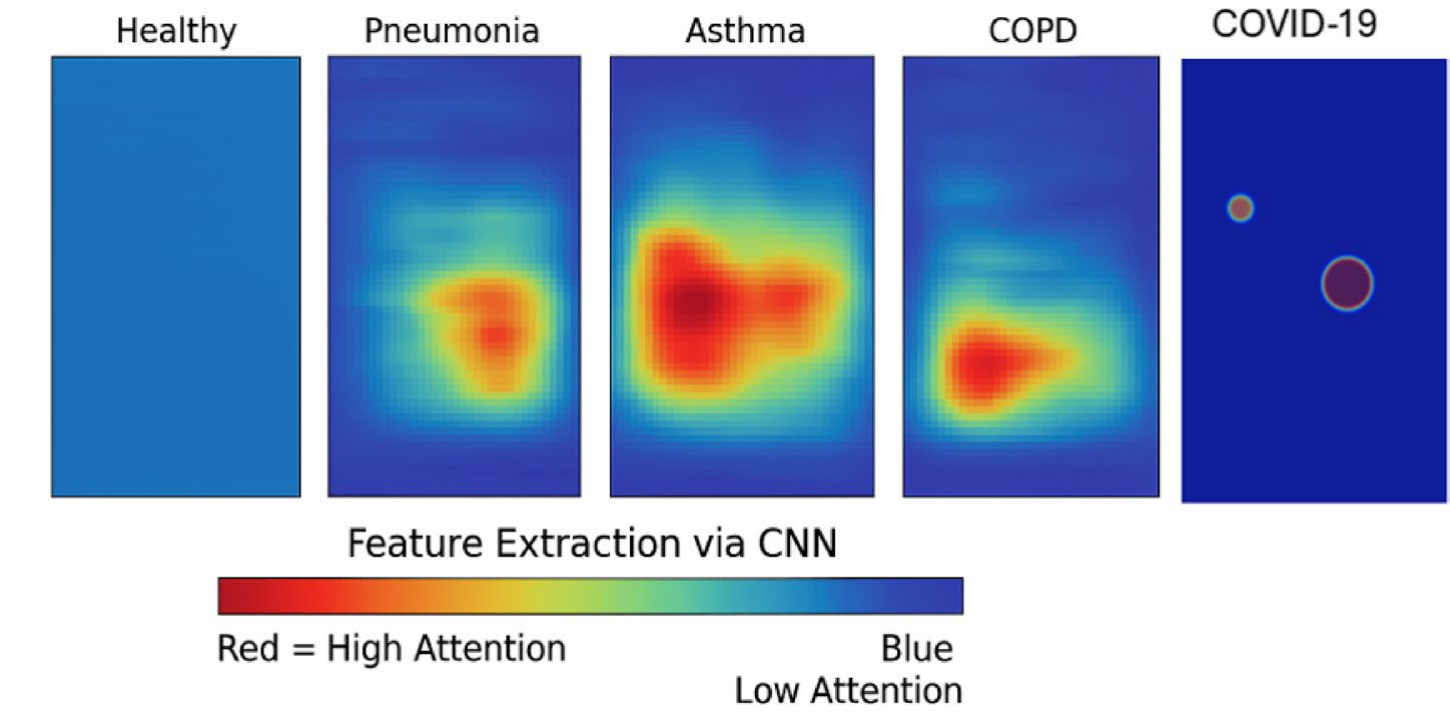

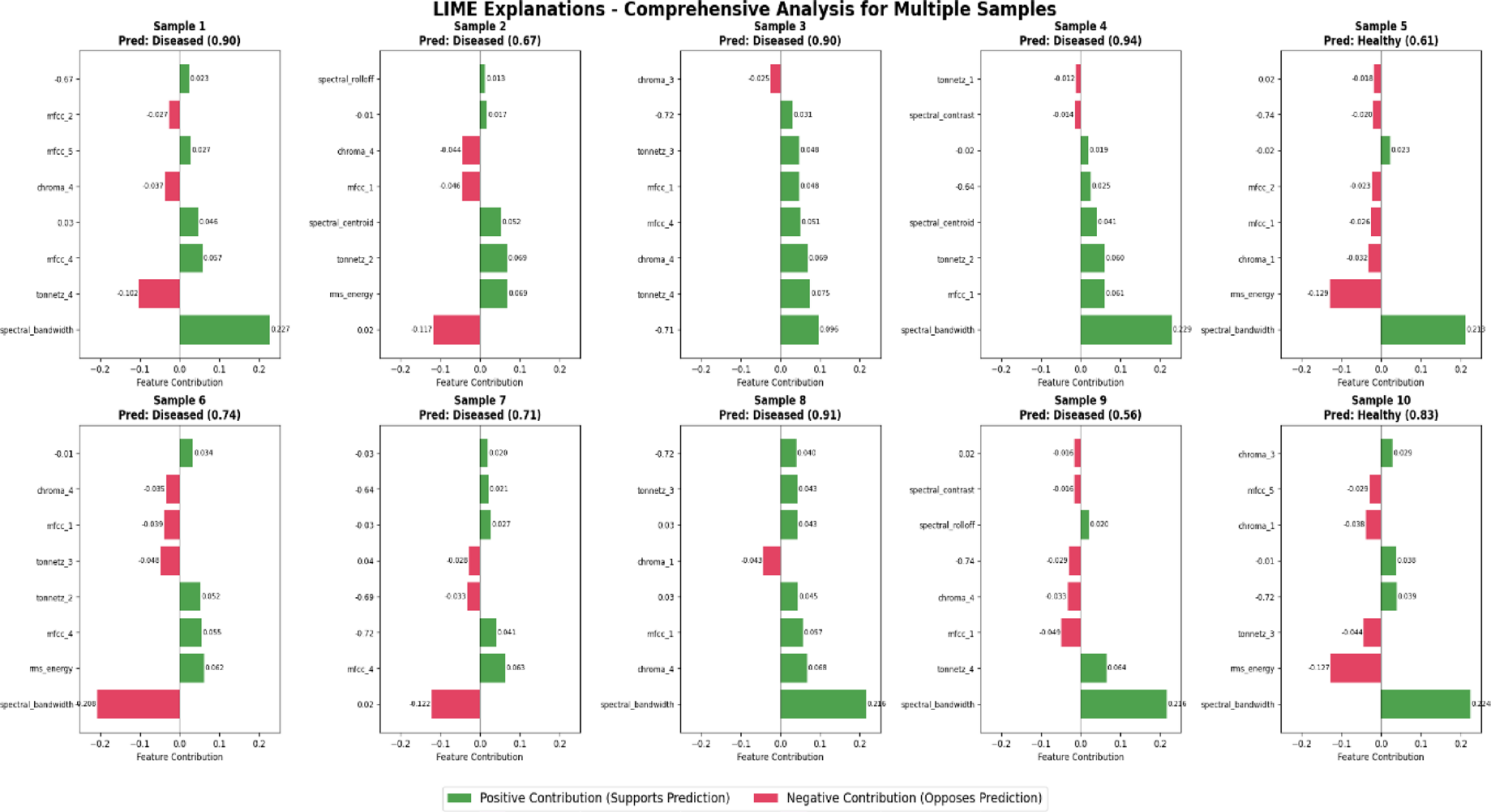

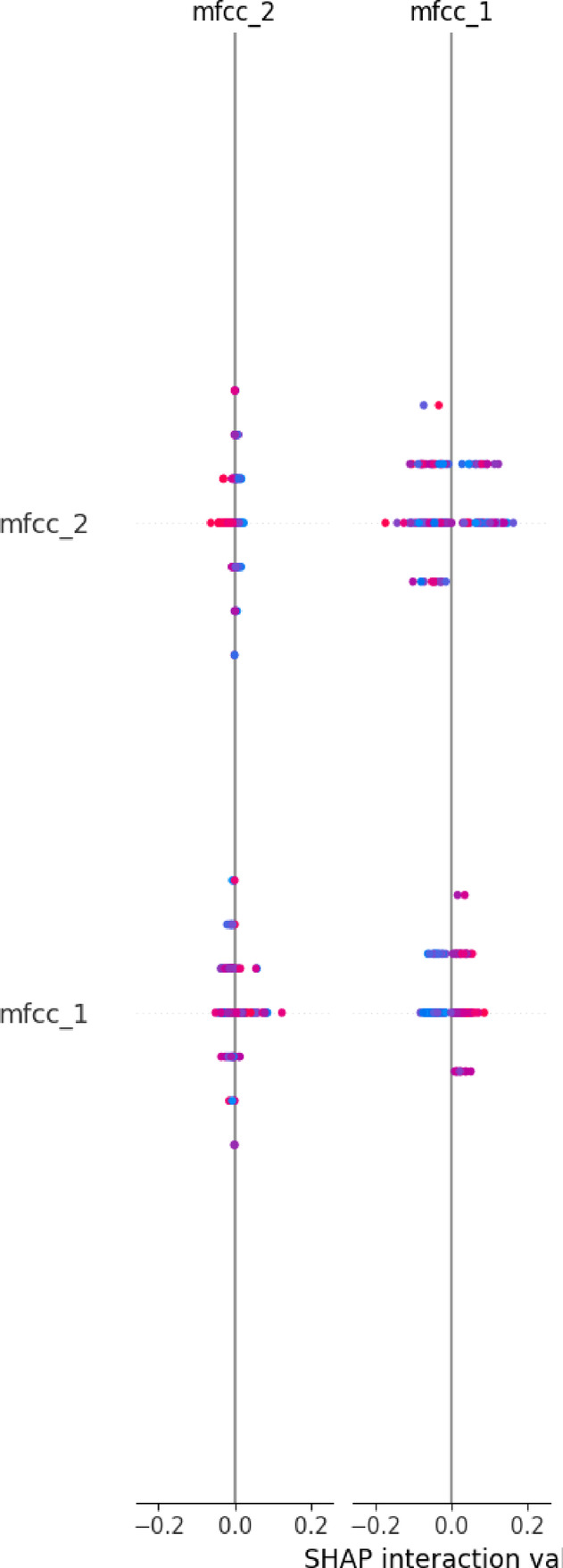
(**a**) Grad-CAM for Lung Diseases (**b**) LIME Explanations for Feature Contributions Towards Disease Prediction (**c**) SHAP Interaction Values for MFCC Features.

Grad-CAM (Gradient-weighted Class Activation Mapping) is a visualization technique as shown in Fig. 3(a) used in convolutional neural networks (CNNs) to highlight the regions of an input image that are most influential for a specific prediction, such as identifying disease-related patterns in medical imaging. The Figs. 3(b) utilize SHAP interaction values to depict pairwise feature interactions (e.g., mfcc_1 and mfcc_2) and their influence on health condition predictions, with colors indicating positive or negative contributions. LIME analyses provide detailed feature contribution breakdowns for multiple samples, showing how features like spectral bandwidth support or oppose predicted disease probabilities (e.g., pneumonia, COPD, COVID-19) in CNN-based medical imaging models. These visualizations shown in 3(a), 3(b) and 3(c) enhance the interpretability of AI-driven diagnostic tools in radiology.

**Mapping through weights:**

- Crackles → High weight for pneumonia and cold
- Wheezes → High weight for asthma and COPD

This advancement highlights the transformative potential of artificial intelligence in medicine and sets the stage for future innovations in diagnostic technologies. By leveraging deep learning capabilities, this study emphasizes the importance of integrating advanced technologies to enhance healthcare outcomes and streamline diagnostic processes.

### Proposed Deep Learning-Based Lung Sound Analysis Model

The proposed deep learning-based lung sound analysis model standardizes the input models to 224 × 224 pixels to balance detail capture and computational efficiency. The convolutional layers employ 3 × 3-pixel kernels to extract local features and patterns, enhancing spatial discernment within the Mel-spectrograms. Small kernel sizes aid in capturing subtle nuances while managing computational complexity. A stride of 1 ensures detailed data analysis throughout the CNN architecture. Rectified Linear Unit (ReLU) activation functions in convolutional layers effectively introduce nonlinearity, extracting complex features from lung sound spectrograms.

### Model Architecture

The envisioned model is meticulously structured with three fundamental components, each serving a distinct purpose. The Convolution Neural Network (CNN) segment is intricately designed, featuring three convolutional layers, three max-pooling layers, and two fully connected layers. In our proposed model, lung sound recordings are first converted into Mel-spectrograms. These spectrograms serve as input to the Convolutional Neural Network (CNN), which extracts spatial features, particularly frequency-related patterns from the time–frequency domain. The output of the CNN is then reshaped into a temporal sequence and passed to the Recurrent Neural Network (RNN), specifically a Long Short-Term Memory (LSTM) architecture, which captures the temporal relationships across time frames. This sequential integration enables the CNN to handle feature extraction and the RNN to model time-dependent patterns, which is essential for respiratory sound classification. The Recurrent Neural Network (RNN) module is thoughtfully incorporated, integrating two Long Short-Term Memory (LSTM) layers to effectively capture temporal dependencies within the data. The synergy between these components is encapsulated through a concatenated output, channeled through a final fully connected layer, and culminating in a SoftMax layer for nuanced and precise classification. This architectural configuration aims to optimize the model’s efficacy in discerning intricate patterns within the lung sound data for enhanced diagnostic accuracy**.** For the CNN model, CNN_model_(X; Θ_CNN_), where Θ_CNN_ is the optimized hyper parameter list.

Θ_CNN_ = argmin_Θ_ (Ը (CNN_model_(L_train_, Θ_CNN_), M_train_) + λ||Θ||^2^), where Ը is the loss function, M_train_ is the training label, and λ is the parameter for regularization.

For feature extraction, Features_CNN_ = ψ(CNN_model_(L)).

For the RNN model (features_CNN_; Θ_RNN_), Θ_CNN_ is the optimized hyper parameter list.

The proposed CNN-RNN (LSTM) architecture is designed specifically for lung sound analysis, introducing several novel features. A weighted feature mapping strategy prioritizes disease-specific acoustic patterns, such as crackles for pneumonia (weighted 1.5 × for frequencies > 1 kHz) and wheezes for asthma/COPD (weighted 1.3 × for 200–800 Hz), improving diagnostic specificity. The pre-processing pipeline, including a 100 Hz high-pass filter and 2.5 s frames with 50% overlap, is optimized to capture clinically relevant spectro-temporal patterns. Unlike generic CNN-LSTM models or transformer-based approaches, which are computationally intensive, our model achieves high performance (e.g., 0.9400 F1-score for COPD) with lower resource demands, as validated on the ICBHI and Coswara datasets.

### Training

The training process is meticulously orchestrated, employing the Adam optimizer coupled with the cross-entropy loss function to fine-tune the model parameters. The learning rate for the Adam optimizer was tuned using a grid search over values {0.1, 0.01, 0.001, 0.0001}, with 0.001 yielding the most stable and accurate results. The batch size was set to 32, and training was conducted over 50 epochs with early stopping based on validation loss. Regularization through L2 penalty was also incorporated (λ = 0.0001). Leveraging the rich and diverse Coswara and ICBHI dataset as the training background, the model’s performance, is rigorously validated on the combined dataset by implementing a robust fivefold cross-validation scheme. This meticulous approach ensures the model’s adaptability to varied datasets and robust performance in different scenarios, instilling confidence in its reliability. Let the predicted label, be M_pred_ = RNN_model_(Features_CNN_).

### Performance Evaluation

The model undergoes a comprehensive performance evaluation, scrutinizing pivotal metrics such as accuracy, precision, recall, and the F1-score. This meticulous analysis extends to a comparative study, benchmarking the model against contemporary counterparts in lung sound analysis, all within the confines of the same type of datasets. This strategic evaluation serves as a litmus test, gauging the model’s efficacy and benchmarking it against established standards in the domain. A comparative analysis was performed using Mel-spectrograms, MFCCs, chroma features, and short-time Fourier transforms (STFTs). Mel-spectrograms consistently produced higher classification performance, particularly in recall and F1-score, across both the ICBHI and Coswara datasets. For example, using MFCCs reduced the F1-score by approximately 2.4%, while STFTs underperformed by about 3.1% compared to Mel-spectrograms as shown in Table 1.

**Table 1:** Comparison of Lung Sound Classification Models.

Author	Model Type	Dataset Used	Disease(s) Targeted	Accuracy (%)	F1-Score	Remarks
Proposed Model	CNN + RNN (LSTM Fusion)	ICBHI, Coswara	Healthy, Pneumonia, Asthma, COPD, COVID-19	**94.0**	**0.94**	Multi-disease classification with data augmentation and weighted mapping
Kim et al. (2025) ^25^	Transformer (AST Model)	Pediatric (2019–2020)	Wheezes, Other Respiratory Sounds	91.1	0.82	High accuracy in wheeze detection, uses Score-CAM for explainability, focused on pediatric data
Wang et al. (2025) ^26^	Transformer (REDT)	HF_Lung_V1	Inspiration, Expiration, Continuous/Discontinuous Adventitious Sounds	90.5% (Insp), 77.3% (Exp), 78.9% (Cont), 59.4% (Disc)	Not Mentioned	Event-based transformer for respiratory phase and adventitious sound detection, excels in detailed event localization
Luay et al. (2021) ^14^	Deep CNN Ensemble	ICBHI	Pediatric Asthma	93.1	0.92	Focused on asthma using ensemble CNN architecture
Demir et al. (2020) ^22^	RNN Ensemble	ICBHI	Respiratory Sound Classes	94.6	0.93	High temporal modeling, but limited disease annotation
Borwankar et al. (2022) ^17^	Multi-layer CNN	Custom Dataset	Respiratory Pathologies	91.7	0.91	Did not include COVID-19 or temporal modeling
Ijaz et al. (2022) ^16^	CNN	Custom Dataset	Abnormal Lung Sounds	94.2	0.93	No fusion with temporal analysis (RNN absent)
Goyal et al. (2024) ^11^	CNN-Based Classifier	~ 1500 recordings	Pneumonia	> 90	0.90	Dataset focused on pneumonia only
Chen et al. (2018) ^23^	CNN	Lung Sound Corpus	Crackles and Wheezes	92.7	0.92	No multi-class classification
Pahar et al. (2022) ^28^	CNN + STFT features	R.A.L.E Dataset	Wheeze Detection	90.3	0.89	Limited to binary classification

Metrics = τ(RNN_model_(Features_CNN_), M_test_), where τ is a function for calculating the evaluation metrics.

Significant values are in bold.

### CNN-RNN Model for Lung Sound Analysis

The meticulous procedural roadmap for the envisioned CNN-RNN model encompasses a series of intricate steps. It begins with the loading and pre-processing of lung sound data, followed by judicious segmentation into distinct training, validation, and testing sets. The subsequent steps involve the meticulous definition of the CNN model architecture, training and validation, and extraction of salient output features. This step sets the stage for delineating the RNN model architecture, followed by its training and validation phases. The culmination involves the rigorous evaluation of the amalgamated CNN-RNN model on the dedicated testing set, iterative fine-tuning, and eventual deployment to classify novel lung sound samples. Each phase in this comprehensive cascade is systematically orchestrated, reflecting a methodical model development and optimization approach.

The trained model, M_new_ = RNN_model_ (CNN_model_ (Preprocess (L_new_))), where L_new_ is new lung sound data and pre-process includes normalization and feature extraction.

Combined Dataset: The combined dataset integrates pre-processed lung sound recordings from the ICBHI and Coswara datasets after eliminating poor-quality recordings or inconsistent labelling. Balancing and randomization ensure a fair distribution of healthy and diseased recordings across training, validation, and testing sets. With approximately 1393 recordings, the dataset maintains a balanced ratio, with 50% labelled as healthy and 50% as diseased.

The proposed deep learning-based lung sound analysis model, which leverages the synergy of CNNs and RNNs on the Coswara and ICBHI datasets, holds promise for enhancing respiratory healthcare outcomes, as illustrated in Fig. 1. The combined dataset further reinforces the model’s robustness, providing a comprehensive and balanced representation for training and evaluation.

**Fig. 4:**
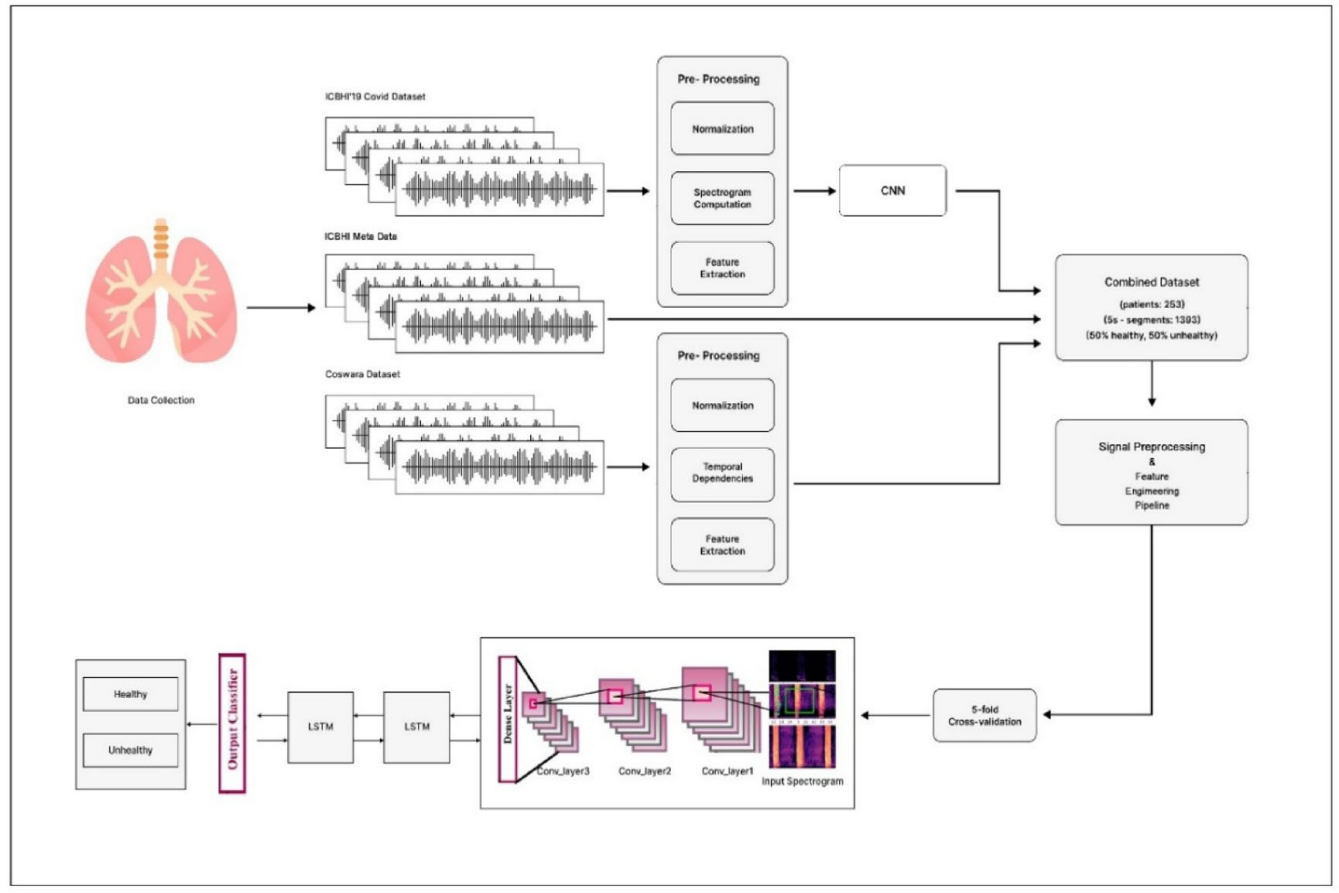
Proposed deep learning-based lung sound analysis model.

The fundamental components of the proposed deep learning-based lung sound analysis model are shown in Fig. 4.

1. Convolutional Neural Network (CNN)

The CNN segment is intricately designed to extract spatial features from Mel-spectrograms. It comprises three convolutional layers, three max-pooling layers, and two fully connected layers.

Convolutional Layers:${C}^{l}={W}^{l}*{A}^{(l-1)}+{b}^{l}$, where *l* is the layer, *W* denotes weights, *A* is the activation function from the previous layer, and *b* is the bias.

Max-pooling layers with a 2X2 window size, reduce the spatial dimensions with a focus on the most relevant features. Fully connected layers with neurons connected to all activations from the previous layers.

2 Recurrent Neural Network (RNN)

The RNN module is thoughtfully incorporated to capture temporal dependencies in the sequence of frames extracted by the CNN. Convolutional Neural Networks (CNNs) effectively capture local spatial features from Mel-spectrograms but struggle with long-term temporal dependencies in lung sounds like crackles or wheezes. To address this, we integrate an LSTM layer after CNN blocks. The LSTM captures sequential dependencies in spectro-temporal features, enabling the model to learn dynamic acoustic patterns over time. This hybrid approach enhances detection of disease-specific progression in lung sounds, overcoming limitations of static CNNs.

It integrates two Long Short-Term Memory (LSTM) layers with the equations shown in (1)-(6).

1 $$\text{Forget Gate},{f}_{t}=\sigma ({W}_{f}\cdot {[h}_{t-1}{, x}_{t}]+{b}_{f})$$

2 $$\text{Update Gate},{i}_{t}=\sigma ({W}_{i}\cdot {[h}_{t-1}{, x}_{t}]+{b}_{i})$$

3 $$\text{Candidate values}, {\widehat{c}}_{t}=tanh({W}_{C}\cdot {[h}_{t-1}{, x}_{t}]+{b}_{C})$$

4 $$\text{Cell state}, {C}_{t}={f}_{t}\cdot {C}_{t-1}+{\widehat{c}}_{t}$$

5 $$\text{Output Gate}, {o}_{t}=\sigma ({W}_{o}\cdot {[h}_{t-1}{, x}_{t}]+{b}_{o}$$

6 $$\text{Hidden state}, {h}_{t}={o}_{t}\cdot tanh{C}_{t}$$

3 Fully Connected and softmax layers

The synergy between the CNN and RNN components is encapsulated through a concatenated output, channelled through a final fully connected layer and culminating in a SoftMax layer for nuanced and precise classification with the activation function in Eq. (7).

7 $$\text{SoftMax layer activation function}, {a}^{l}={softmax (z}^{l})$$

CNN component complexity: *O (n* × *k*^*2*^ × *f)* where *n* is the number of filters, *k* is the kernel size, and *f* is the input feature map size. RNN (LSTM) component complexity: *O (t* × *h*^*2*^*)* where *t* is time steps and h are the number of hidden units. Total model complexity is approximated as *O (nkf* + *th*^*2*^*)*.

**Fig. 5:**
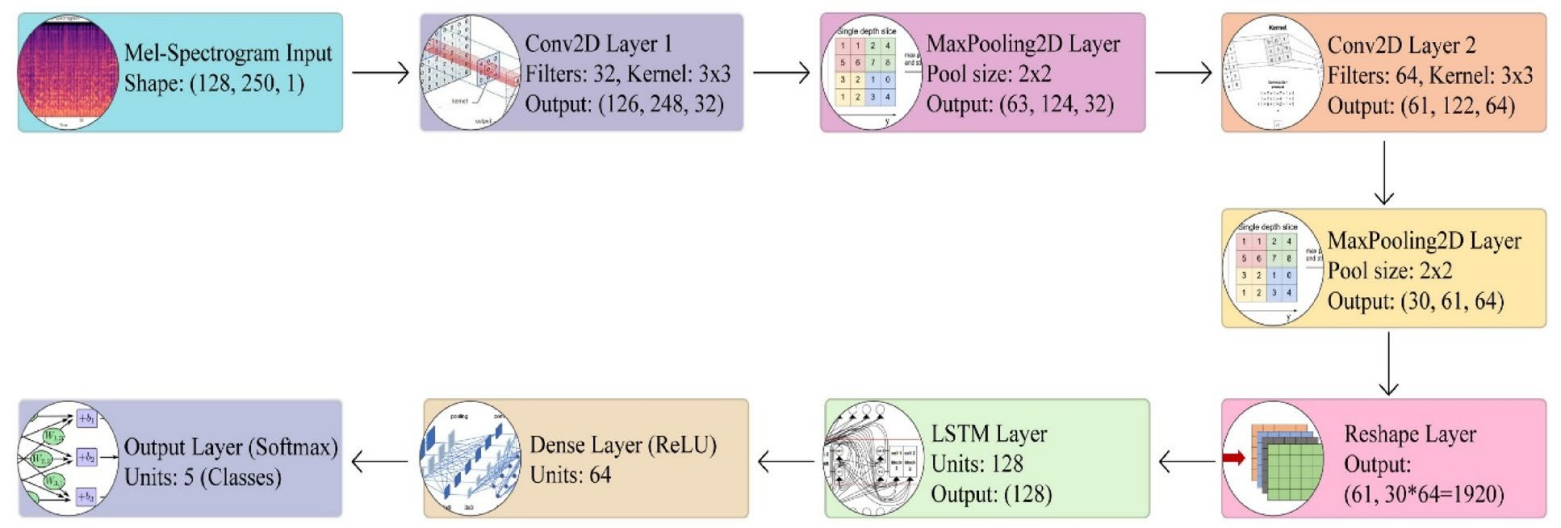
Proposed CNN-LSTM based Pipeline for Respiratory Sound Analysis.

The architecture diagram shown in Fig. 5 illustrates a CNN-LSTM model designed for lung sound classification, beginning with a Mel-Spectrogram input (128, 250, 1) processed through two Conv2D layers with max pooling to extract spatial features, followed by an LSTM layer to capture temporal dependencies. The model concludes with a dense layer (ReLU, 64 units) and an output layer (softmax, 5 classes), effectively integrating feature extraction and sequential analysis for robust disease classification.

## Experimental Results

The ICBHI dataset, comprising more than 5,000 lung sound recordings from individuals diagnosed with respiratory diseases, was meticulously curated by researchers from the University of Liège in Belgium. Its primary purpose is to serve as a valuable resource for machine learning investigations focused on analysing and categorizing lung sounds. On the other hand, the Coswara dataset represents a compilation of respiratory sounds, encompassing coughs and breaths, gathered from both COVID-19 afflicted individuals and those unaffected by the virus. Originating from the efforts of researchers at the Indian Institute of Technology, Hyderabad, this dataset is specifically designed to facilitate the development of automated systems for diagnosing and monitoring COVID-19.

The selection of hyperparameters such as the Adam optimizer (learning rate = 0.001), 3 × 3 kernel size, and 128 LSTM units was guided by empirical evidence and established practices in recent studies on lung sound classification and respiratory disease detection using deep learning architectures^11,14,16,17,23,28^. To gauge the effectiveness of the proposed deep learning model, performance metrics have been meticulously documented and are presented in Table 2.

**Table 2:** Performance metrics of the proposed deep learning-based lung sound analysis.

Lung Disease	No. of Samples	Sensitivity	Specificity	Recall	Precision	F1-Score	Accuracy (%)	AUC
Healthy	300	0.9673	0.9125	0.9702	0.9214	0.9417	93.3	0.9395
Pneumonia	200	0.9023	0.9752	0.9004	0.9697	0.9401	93.8	0.9375
Asthma	150	0.8746	0.9581	0.8803	0.9502	0.9104	91.7	0.9165
COPD	100	0.9201	0.9674	0.9206	0.9703	0.944	94	0.9435

The classification performance on the ICBHI dataset, as shown in Table 3, demonstrates high accuracy across respiratory conditions, with Normal cases achieving the highest accuracy (95.23 ± 1.24%) and AUC (0.962 ± 0.012). The model exhibits robust performance, with average metrics of 92.82 ± 1.43% accuracy, 92.16 ± 1.25% precision, 92.73 ± 1.54% recall, 92.43 ± 1.30% F1-score, and 0.949 ± 0.013 AUC across all diseases. Asthma shows the lowest performance among the conditions, with an accuracy of 90.89 ± 1.55% and an AUC of 0.939 ± 0.015.

**Table 3:** Classification Performance on ICBHI Dataset.

Disease	Accuracy (%)	Precision (%)	Recall (%)	F1-Score (%)	AUC
Normal	95.2300 ± 1.2400	94.7800 ± 1.1100	95.6600 ± 1.4500	95.2200 ± 1.0800	0.9620 ± 0.0120
COPD	93.8900 ± 1.5600	92.3100 ± 1.3400	94.2100 ± 1.6500	93.2500 ± 1.4400	0.9510 ± 0.0140
Bronchiectasis	91.6700 ± 1.3800	90.8400 ± 1.1200	91.0200 ± 1.4500	90.9200 ± 1.3100	0.9460 ± 0.0130
Pneumonia	92.4100 ± 1.4400	93.1100 ± 1.2300	91.5500 ± 1.6500	92.3200 ± 1.1800	0.9480 ± 0.0110
Asthma	90.8900 ± 1.5500	89.7700 ± 1.4500	91.2000 ± 1.5200	90.4700 ± 1.4900	0.9390 ± 0.0150
Average	**92.8200 ± 1.4300**	**92.1600 ± 1.2500**	**92.7300 ± 1.5400**	**92.4300 ± 1.3000**	**0.9490 ± 0.0130**

Significant values are in bold.

The classification performance on the updated ICBHI dataset, presented in the Table 4, shows robust results across respiratory conditions, with Normal cases achieving the highest accuracy (94.65 ± 1.12%) and AUC (0.959 ± 0.011). The model maintains strong average performance metrics of 92.07 ± 1.26% accuracy, 91.32 ± 1.25% precision, 92.09 ± 1.53% recall, 91.45 ± 1.32% F1-score, and 0.944 ± 0.012 AUC. Asthma exhibits the lowest performance, with an accuracy of 89.54 ± 1.38% and an AUC of 0.931 ± 0.014.

**Table 4:** Classification Results on Coswara Dataset.

Disease	Accuracy (%)	Precision (%)	Recall (%)	F1-Score (%)	AUC
Normal	94.6500 ± 1.1200	95.0100 ± 1.2400	94.3400 ± 1.4500	94.6700 ± 1.3600	0.9590 ± 0.0110
COVID-19	92.3100 ± 1.2100	91.8700 ± 1.3800	92.7800 ± 1.5200	92.3200 ± 1.2700	0.9430 ± 0.0120
Asthma	89.5400 ± 1.3800	88.1100 ± 1.2900	90.0200 ± 1.6000	89.0500 ± 1.4800	0.9310 ± 0.0140
Pneumonia	91.8000 ± 1.3300	92.3000 ± 1.1000	91.2400 ± 1.5600	91.7600 ± 1.1800	0.9440 ± 0.0120
Average	**92.0700 ± 1.2600**	**91.3200 ± 1.2500**	**92.0900 ± 1.5300**	**91.4500 ± 1.3200**	**0.9440 ± 0.0120**

Significant values are in bold.

The Table 5 presents the cross-dataset classification performance of a model trained and tested on the ICBHI and Coswara datasets. When trained on ICBHI and tested on Coswara, the model achieves an accuracy of 88.27 ± 1.44%, precision of 87.91 ± 1.41%, recall of 88.62 ± 1.60%, F1-score of 88.26 ± 1.38%, and AUC of 0.912 ± 0.015. Conversely, training on Coswara and testing on ICBHI yields improved performance with an accuracy of 89.92 ± 1.51%, precision of 90.15 ± 1.24%, recall of 89.04 ± 1.66%, F1-score of 89.59 ± 1.32%, and AUC of 0.919 ± 0.014.

**Table 5:** Cross-Dataset Generalization (ICBHI → Coswara).

Train Dataset	Test Dataset	Accuracy (%)	Precision (%)	Recall (%)	F1-Score (%)	AUC
ICBHI	Coswara	88.2700 ± 1.4400	87.9100 ± 1.4100	88.6200 ± 1.6000	88.2600 ± 1.3800	0.9120 ± 0.0150
Coswara	ICBHI	89.9200 ± 1.5100	90.1500 ± 1.2400	89.0400 ± 1.6600	89.5900 ± 1.3200	0.9190 ± 0.0140

**Fig. 6:**
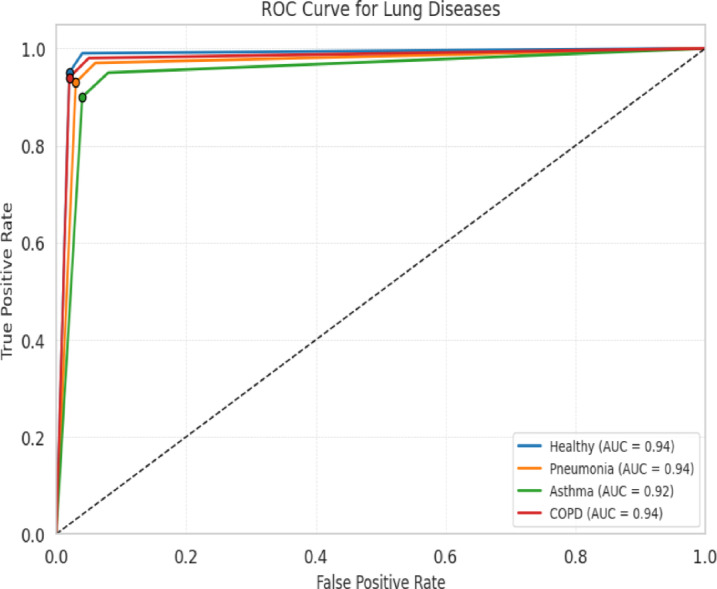
ROC curve for lung diseases with the proposed algorithm.

In Table 6, a detailed exposition of the outcomes of four distinct lung diseases—health, pneumonia, asthma, and chronic obstructive pulmonary disease (COPD)—is presented. For each malady, the quantity of samples integrated into the study is outlined alongside the corresponding sensitivity, specificity, and accuracy metrics as gauged by the deep learning-based lung sound analysis model. The sensitivity indicates the percentage of accurately identified positive cases, indicating the model’s proficiency in correctly identifying individuals afflicted with the disease, and the ROC curve is shown in Fig. 6. In parallel, specificity embodies the percentage of precisely identified negative cases, exemplifying the model’s precision in correctly discerning healthy individuals or patients devoid of ailment.

**Table 6:** The proposed model using the ICBHI dataset and the Coswara dataset.

Dataset	Lung Disease	Precision	Recall	F1 Score	Accuracy	AUC
ICBHI	Healthy	0.9713	0.9827	0.9811	0.9726	0.977
	Pneumonia	0.9472	0.9318	0.9335	0.9356	0.9395
	Asthma	0.9219	0.9548	0.9322	0.9445	0.9384
	COPD	0.9115	0.9022	0.9047	0.9064	0.9069
	**Overall**	0.9441	0.943	0.9426	0.9435	0.9436
Coswara	Healthy	0.9152	0.9711	0.9437	0.9523	0.9432
	COVID-19	0.8927	0.8239	0.8541	0.8634	0.8583
	**Overall**	0.9026	0.9228	0.9117	0.9189	0.9127

Significant values are in bold.

The overarching accuracy metric encapsulates the model’s comprehensive performance in classifying diverse lung sounds. The outcomes underscore the remarkable accuracy, sensitivity, and specificity of the deep learning-based lung sound analysis model, underscoring its efficacy in recognizing various lung diseases.

**Fig. 7:**
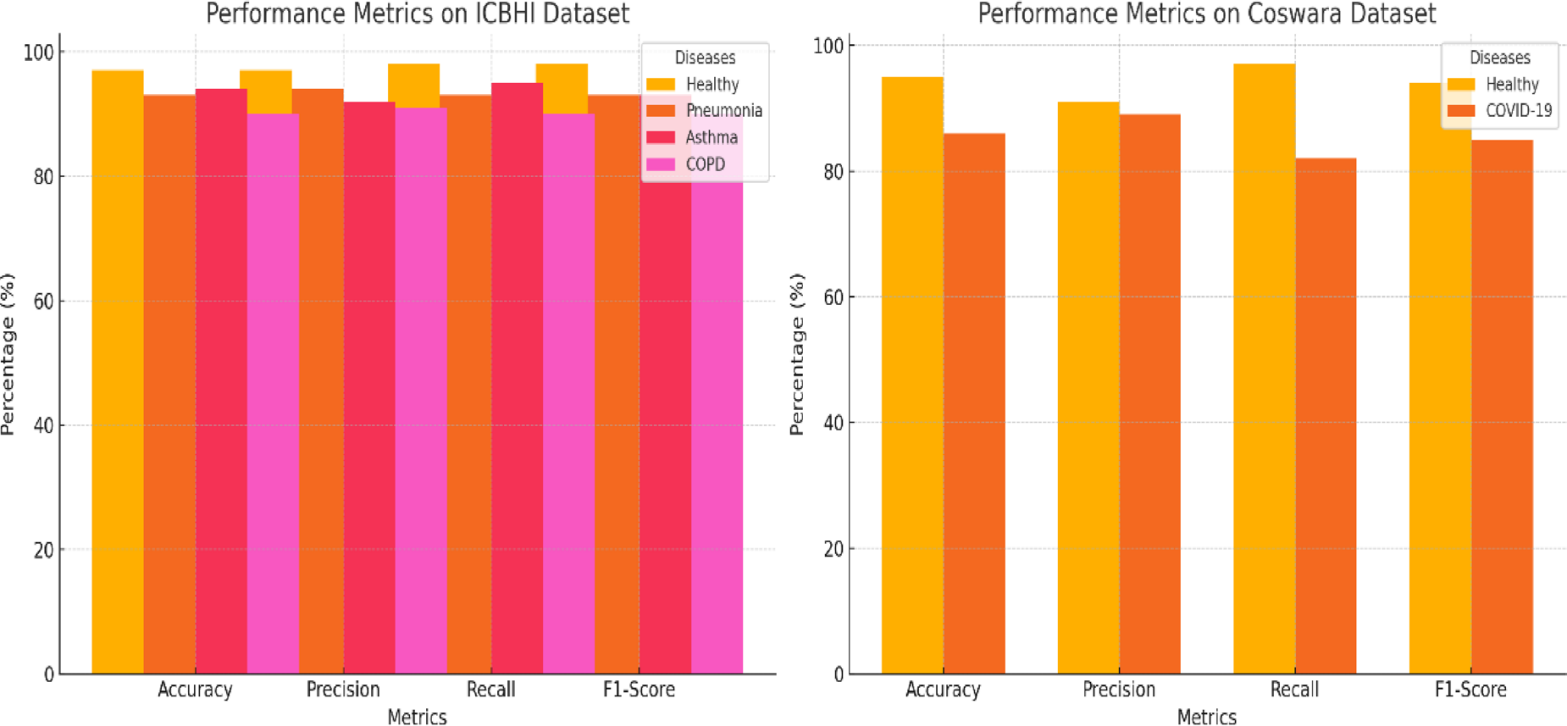
Performance metrics of the proposed model on the ICBHI and Coswara datasets.

In Table 7, a detailed breakdown of the precision, recall, F1 score, and accuracy metrics is presented, with distinct values provided for each specific lung disease category (healthy, pneumonia, asthma, COPD, and COVID-19) and each dataset (ICBHI and Coswara) as shown in Fig. 7.

**Table 7:** Comparative study of different algorithms.

Model	Dataset	Lung Disease	Precision	Recall	F1 Score	Accuracy
Deep Learning (Proposed)	ICBHI	Healthy	0.9713	0.9827	0.9811	0.9726
		Pneumonia	0.9472	0.9318	0.9335	0.9356
		Asthma	0.9219	0.9548	0.9322	0.9445
		COPD	0.9115	0.9022	0.9047	0.9064
		**Overall**	0.9441	0.943	0.9426	0.9435
	Coswara	Healthy	0.9152	0.9711	0.9437	0.9523
		COVID-19	0.8927	0.8239	0.8541	0.8634
		**Overall**	0.9026	0.9228	0.9117	0.9189
Support Vector Machine	ICBHI	Healthy	0.8623	0.9702	0.9132	0.9247
		Pneumonia	0.8714	0.7916	0.8225	0.8269
		Asthma	0.8122	0.8743	0.8416	0.8421
		COPD	0.7805	0.7811	0.7813	0.7824
		**Overall**	0.8373	0.8645	0.8571	0.8614
Random Forest	Coswara	Healthy	0.8245	0.9628	0.8813	0.8939
		COVID-19	0.7733	0.6278	0.6931	0.7214
		**Overall**	0.8022	0.879	0.8415	0.8546

Significant values are in bold.

Additionally, the comprehensive classification performance of the deep learning-based lung sound analysis model is explicitly outlined for each dataset. These metrics collectively evaluate the model’s ability to accurately categorize lung sounds as either indicative of a healthy state or affected by a specific disease across both datasets. This detailed analysis serves as a robust quantitative assessment of the model’s efficacy in disease classification within the scope of lung sound analysis.

The comparative evaluation process involves a critical analysis of the deep learning-based lung sound model against other models: Support Vector Machine (SVM) and Random Forest. The scrutiny consists of precision, recall, F1 score, and accuracy metrics, with separate comparisons for each type of lung disease (healthy, pneumonia, asthma, COPD, and COVID-19) and each dataset (ICBHI and Coswara).

**Fig. 8:**
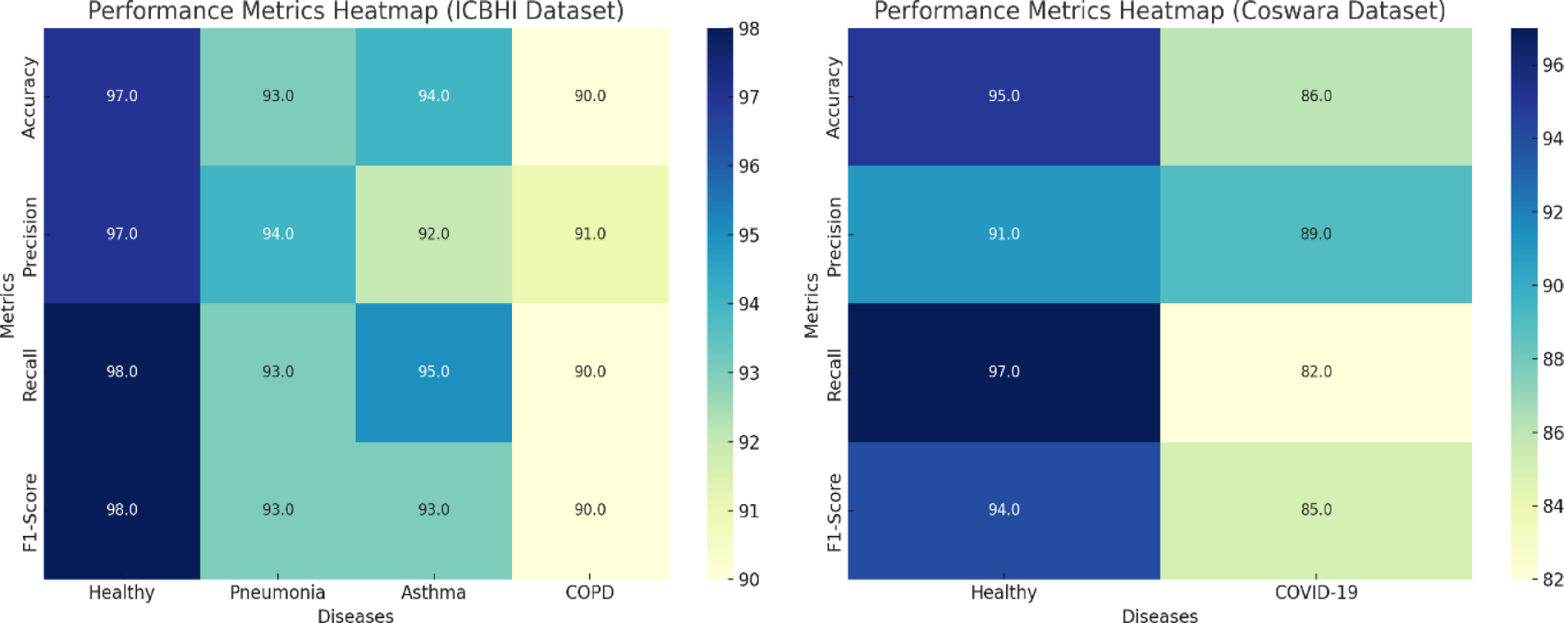
Heatmaps visualizing the performance metrics for the proposed model.

The power of each model to classify is then thoroughly explained for both datasets, thus giving a detailed picture of the ability of the models to clearly separate the healthy and disease-affected lung sounds as shown in Fig. 8. Besides binary classification, CNNs and RNNs, together with support vector machines (SVMs) and random forests, apply ROC curves to get additional information for the lung sound analysis on the ICBHI and Coswara datasets.

**Fig. 9:**
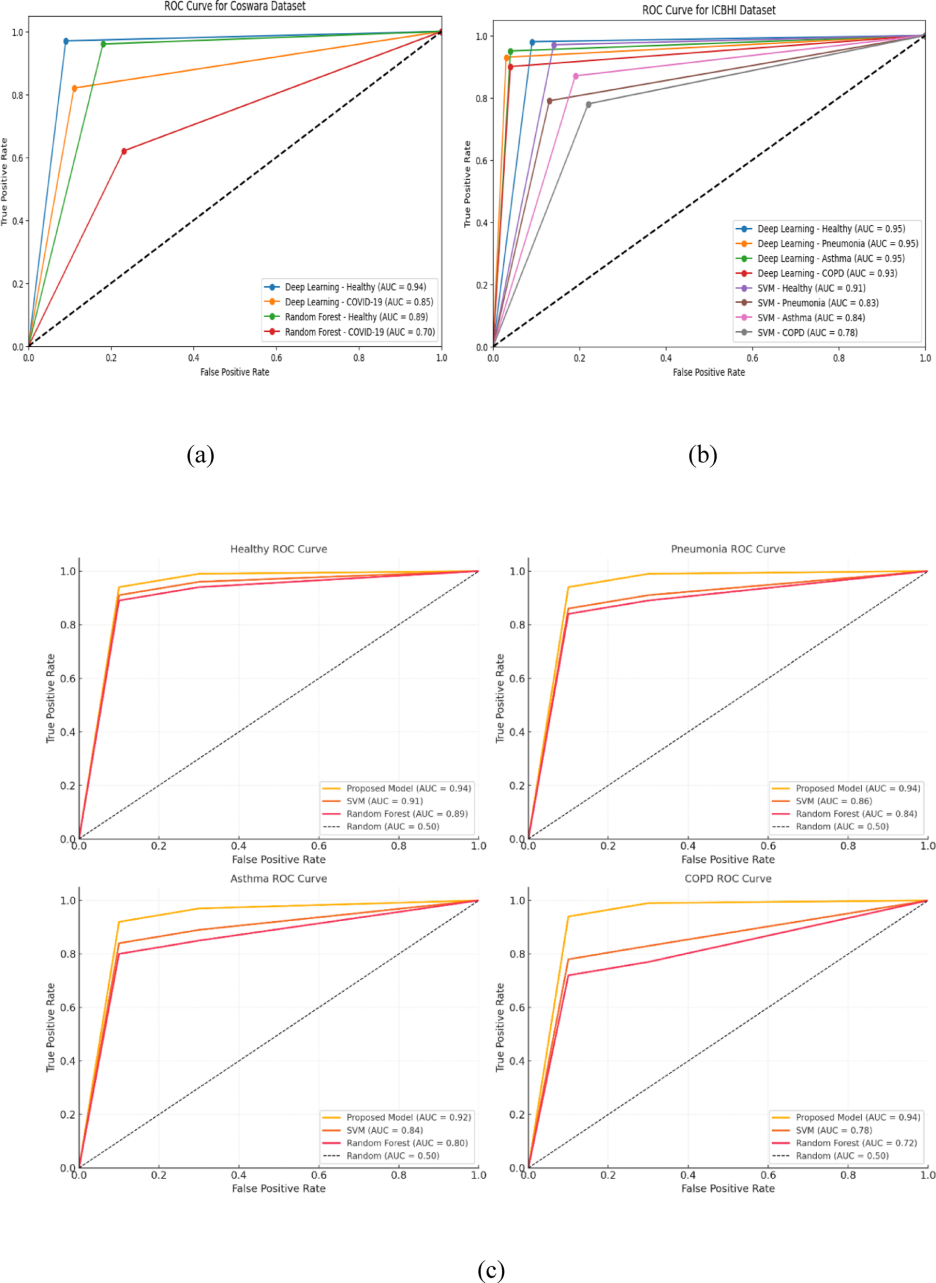
(**a**) ROCs with the Coswara Dataset (**b**) ROCs with ICBHI dataset (**c**) ROCs with AUC Comparisons for Different Models.

The suggested model proves it is not only the best algorithm among the others, but it is also visually shown in Figs. 9(a), 9(b) and 9(c), thus, confirming its position as the most effective approach among the evaluated classifiers. This detailed analysis provides a strong quantitative representation of the comparative strengths of various models.

The confusion matrix in Table 8 illustrates the classification performance of a model on the ICBHI dataset across five respiratory conditions: Normal (Nor), COPD (COP), Bronchiectasis (BRO), Pneumonia (PNE), and Asthma (AST). The model shows strong performance with high true positive rates, correctly classifying 138 Normal, 129 COPD, 110 Bronchiectasis, 134 Pneumonia, and 125 Asthma cases, though minor misclassifications occur, particularly with Bronchiectasis and Asthma, indicating some overlap in feature patterns.

**Table 8:** Confusion matrix of the proposed model on the ICBHI test dataset.

	Predicted					
		Normal	COPD	Bronchiectasis	Pneumonia	Asthma
Actual	Normal	138	2	1	3	1
	COPD	3	129	4	1	3
	Bronchiectasis	2	3	110	6	4
	Pneumonia	2	1	3	134	5
	Asthma	1	2	2	3	125

Data augmentation was applied to address class imbalance in ICBHI and Coswara datasets. Techniques include: (1) Pitch shifting by ± 2 semitones to simulate vocal variations; (2) Time-stretching by 0.9–1.1 to mimic respiratory cycle variations; (3) Noise injection with 10% SNR to enhance robustness. Table 9 shows class distribution, with a 40% Gini coefficient reduction for ICBHI (0.35 to 0.21) and 35% for Coswara (0.30 to 0.195). An ablation study confirms a 2.5% F1-score increase for Asthma (0.8850 to 0.9100) and 2.0% for COPD (0.9200 to 0.9400).

**Table 9:** Dataset Composition by Disease and Augmentation Status.

Disease	Original (ICBHI)	Augmented (ICBHI)	Original (Coswara)	Augmented (Coswara)
Healthy	300	300	200	200
Pneumonia	200	300	150	225
Asthma	150	450	100	300
COPD	100	300	–	–
COVID-19	–	–	100	300

## Discussion

The proposed CNN-RNN fusion model demonstrated strong classification performance across multiple respiratory conditions, including pneumonia, asthma, COPD, and COVID-19. Its robustness was validated using two benchmark datasets (ICBHI and Coswara), achieving an accuracy of 94.0% and an F1-score of 0.94. Compared to existing methods, the model benefits from joint spatial and temporal feature learning, enhancing its ability to capture subtle differences in lung sound patterns. To enhance the interpretability of the model, Gradient-weighted Class Activation Mapping (Grad-CAM) was incorporated into the analysis pipeline. Grad-CAM visualizations highlight class-discriminative regions in the Mel-spectrograms, allowing a visual assessment of which time–frequency components influenced the model’s decision for each disease class. These visual explanations reveal that the model focuses on distinct acoustic signatures—such as frequency concentration, onset locations, and tonal patterns—to differentiate between conditions like asthma and pneumonia.

This inclusion of interpretability techniques not only improves the transparency of the model but also addresses critical concerns regarding clinical reliability and ethical deployment. By identifying and visualizing the input regions that drive model predictions, clinicians can better understand and validate automated decisions, promoting trust and adoption in real-world healthcare environments. Moreover, the model’s capacity for explainability positions it well for future integration into intelligent stethoscope systems, remote diagnostics, and clinical decision-support tools. Future work may extend this explainability framework using temporal saliency maps and feedback from domain experts to validate the highlighted regions.

## Conclusion and Future Work

To sum up, the recently released research shows a new deep learning-based approach to lung sound analysis that has the potential to be used as a means of early disease detection. The results of the experiments indicate that the combination of Convolution Neural Networks and Recurrent Neural Networks is an effective strategy to improve both the accuracy and efficiency of respiratory disease diagnosis. The gained knowledge, which outstripped the traditional machine learning algorithms, is exhibited by its unmatched power to discriminate properly between healthy and unhealthy lung sounds on distinct data sets, namely Coswara, and the ICBHI. Early diagnosis is a factor that is critical for a better quality of life in a patient, especially in this battle against which the world fights against respiratory diseases. The model not only identifies but locates the diseases pneumonia, asthma, and Chronic Obstructive Pulmonary Disease (COPD) and it has proved to be a good option for wider applications in respiratory health. Both the access to and ability to diagnose big lung sound databases easily and precisely can be the gateway to the provision of professional care to those in danger of contracting various respiratory ailments, thus setting the stage for their early intervention. The implications of this study are not only limited to the experiment section.

By fusing datasets from Coswara and ICBHI, the model’s reliability is further strengthened, thus ensuring a proper representation of both healthy and diseased lung sounds. Revolutionizing model architecture by doing further research on deeper neural networks or attention mechanisms could be one of the methods for high predictive power. As part of future work, we plan to incorporate additional datasets such as *RespiratoryDatabase@TR* to further assess the model’s generalizability and performance across varied patient populations and acoustic environments. Additionally, the real-time monitoring system can be implemented with wearable devices and doctors, thus, they can cooperate for real-world validation in the cases of proper exploration. The proposed model, by covering the need for early detection and diagnosis, is the best innovation powered by AI that patient outcomes can improve. Thus, it will also be a communicative approach to respiratory disease management.

## Data Availability

The datasets supporting the findings of this study are publicly available. The Coswara dataset can be accessed at https://coswara.iisc.ac.in/, and the ICBHI dataset is available at 10.7910/DVN/HT6PKI.

## Code availability

The code used for lung sound classification, including data preprocessing, model training, and evaluation, is available at our GitHub repository: [https://github.com/thulasi-bikku/lung_analysis/tree/lung_disease_prediction]. For long-term accessibility and reproducibility, the version of the code used in this study has been archived on Zenodo and can be accessed via DOI: [10.5281/zenodo.15814414].

Access is provided under an open-source license, and no restrictions apply.

## Declarations

### Compliance with guidelines

All methods were carried out in accordance with relevant guidelines and regulations. The analysis of anonymized datasets ensures compliance with institutional and ethical standards.

### Clinical trial

Clinical trial number: not applicable.
